# Comparison of the intubation success rate between the intubating catheter and videolaryngoscope in difficult airways: a prospective randomized trial

**DOI:** 10.1016/j.bjane.2021.04.027

**Published:** 2021-05-12

**Authors:** Aysun Ozdemirkan, Ozkan Onal, Irem Gumus Ozcan, Emine Aslanlar, Ali Saltali, Mehmet Sari, Cansu Ciftci, Hasan Huseyin Bayram

**Affiliations:** a Selcuk University Faculty of Medicine, Department of Anesthesiology and Intensive Care, Konya, Turkey; b Cleveland Clinic Main Hospital, Anesthesiology Institute, Department of Outcomes Research, Cleveland, Ohio, USA

**Keywords:** Airway management, Catheters, Equipment and supplies, Intubation, Intratracheal

## Abstract

**Background:**

Several devices and algorithms have already been examined and compared for difficult airway management. However, there is no existing study comparing the success of the Intubating Catheter (IC) and the Videolaryngoscope (VL) in patients who are difficult to intubate. We aimed to compare Frova IC and McGrath VL in terms of intubation success rates in patients with difficult intubation.

**Methods:**

This prospective, randomized study was performed in an university hospital. Patients who underwent an operation under general anesthesia and whom airway management process was deemed difficult were included in this study. Patients were randomly divided into two groups by envelopes containing a number: the intubating catheter group (Group IC), intubated using the Frova IC, and the videolaryngoscope group (Group VL), intubated using the McGrath VL. Study data were collected by a technician who was blind to the study groups and the type of device used in the intubation procedure.

**Results:**

A total of 49 patients with difficult airway were included in the study, including 25 patients in the Frova IC Group and 24 patients in the McGrath VL Group. The rate of successful intubation was determined to be 88% in Group IC and 66% in Group VL (*p* = 0.074). The mean duration of intubation attempt in Group VL was 44.62 seconds, whereas in Group IC, it was 51.12 seconds (*p* = 0.593). Group VL was found to have a significantly lower Cormack-Lehane grade compared to Group IC (*p* < 0.001).

**Conclusion:**

Frova IC is a candidate to be an indispensable instrument in terms of cost-effectiveness in clinics such as anesthesia and emergency medicine, where difficult intubation cases are frequently encountered. However, the combination of Frova IC and McGrath VL seems to be more successful in difficult intubation situations, so future studies should focus on using these two devices together.

## Introduction

Videolaryngoscopes (VLs) have been increasingly used for patients with difficult airways.^1^ These can simplify tracheal intubation by supplying high-quality image of the larynx without the setting of the three-airway axes (oral-pharyngeal-laryngeal).^2^ VLs has been found to reduce problems associated with intubation by allowing the remote observation of the glottic opening, and its use has been seen to improve the Cormack-Lehane (CL) grade. CL grade classifies the views obtained via direct laryngoscopy based on the structures seen.^3^ On the other hand, using VLs has been identified to improve the overall success ratio of intubations^4^, ^5^ compared with direct laryngoscopy; they can also be used to teach beginning practitioners.^6^ An example is the McGrath VL, which has been defined as a movable VL with constricted-angulated single-use blades.^7^ However, the straight laryngeal image provided by VLs does not warrant quicker or successful intubation every time, and, for this reason, different VLs have been examined.^8^

Intubating devices such as bougies and stylets have been determined to improve intubation in difficult airways; furthermore, they are relatively cheap, light, and handy, increasing the probability of successful intubation in a restricted glottic appearance.^9^ Meanwhile, the Frova Intubating Catheter (IC) introduced in 1988 is a gum-elastic bougie meant to improve endotracheal intubation. It may help overcome some of the restrictions of VLs, offering various benefits.^10^ When utilized together with a VL, the Frova IC can fix some issues in terms VL use.^11^

In previous studies, many devices used in difficult intubations have been compared using a manikin.^12^, ^13^, ^14^ In studies carried out in clinics, patients who are not candidates for difficult intubation are largely preferred. The number of studies on patients who are deemed difficult to intubate is quite low.^15^, ^16^ It is known that many factors may predispose patients for the condition known as difficult intubation, and these factors can be detected beforehand. In order to cope with this problem, a number of materials, devices, and strategies have been developed. But, as far as we know, there is no existing study comparing the success of IC and VL on patients who are difficult to intubate. We planned our study because there is none on this subject in the literature.

Our primary outcome was to compare the intubation success rate of Frova IC with the success rate of McGrath VL in adult patients with difficult intubation.

## Methods

### Study Design

This prospective, randomized, single-blind, parallel-group study was approved by the Selcuk University Faculty of Medicine Research Ethics Committee (Konya, Turkey, numbered 050.01.04). Informed consent was obtained from all patients. Our study was supported by the Selcuk University Scientific Research Projects Coordination Unit (Project number: 15102023). The research carried out in accordance with Declaration of Helsinki for experiments involving humans.

### Participants and intervention groups

In total, 49 patients with an American Society of Anesthesiologists (ASA) physical status I–III and aged 18–65 years who were undergoing elective surgical procedures requiring tracheal intubation in a university hospital were included in this study. Patients who were pregnant, emergency cases, and younger than 18 years and with severe pulmonary disease were excluded. Patients who failed in at least one intubation attempt (including stylet use, changing the number of blades, repositioning) by an anesthetist with at least 3 years of experience and identified as difficult to intubate during laryngoscopy were included in this study. Patients were randomly assigned to one of two parallel groups, in 1:1 ratio in terms of the device used for intubation. Patients were grouped into one of the two study groups. Group IC (n = 25) consisted of patients who were intubated using Frova IC (Frova Intubating Introducer, Cook Limited, Letchworth, Hertfordshire, UK) via the Macintosh laryngoscope (size 3 blade in females: size 4 in males). Meanwhile, Group VL (n = 24) consisted of patients intubated using the McGrath VL (Aircraft Medical, Edinburgh, UK).

### Anesthesia induction procedure

All cases received a uniform general anesthetic. Usual monitoring involved electrocardiogram, noninvasive arterial pressure, SpO_2_, and evaluation of end-tidal carbon dioxide and volatile anesthetic amounts. All cases were preoxygenated using a facemask capable of reaching a fractional O_2_ of leastways 0.8 prior to the induction of anesthesia. General anesthesia was carried with propofol 1–3 mg.kg^-1^ (Propofol, Fresenius, Istanbul, Turkey) and 0.5–1 mg.kg^-1^ rocuronium (Esmeron, Organon, Istanbul, Turkey). Then, fentanyl 2 mcg.kg^-1^ (Talinat, Istanbul, Turkey) and remifentanil 0.5–1 mcg.kg^-1^.min^-1^ (Ultiva, GlaxoSmithKline, Istanbul, Turkey) was applied.

Loss of perception was then achieved, facemask ventilation was started, and the anesthesia was provided by a volatile anesthetic factor (with an age-set minimum alveolar concentration of 1.0). Two minutes after the application of neuromuscular block, laryngoscopy was started by one of four anesthetists (AO, OO, EA and IG) who are skilled in the use of either instrument. Because of the brevity of this 2-minute period, the sufficiency of neuromuscular block prior to intubation was not formally counted. Every anesthetist had applied more than 500 intubations using the Macintosh laryngoscope and at least 50 intubations using the Frova IC and the VL (McGrath) on patients prior to this study.

### Endotracheal intubation procedure

In Group IC, the single-use Frova IC was employed. The Macintosh laryngoscope was then placed into the mouth within the median line and was forwarded gently above the tongue throughout the palatal wall until the epiglottis came to view. The blade was at the same time forwarded below the epiglottis until the glottis was viewed, and Frova IC was then passed through below the epiglottis, and the tip of the catheter was advanced toward the glottis. After the sensation and transition from the tracheal ring and 2–3 cm of introduction, the stiffening cannula was removed. While the position of the Frova IC was maintained, the endotracheal tube was advanced to an appropriate distance to the trachea, and Frova IC was then gently removed.

In Group VL, McGrath VL was employed. The endotracheal tube was carefully guided directly to the glottis and passed between the vocal cords.

In cases when intubations could not be carried out using the chosen device at the third attempt by an anesthetist with at least 3 years of experience, the device was considered to be unsuccessful; thus, the second device will be utilized. When this method still proved unsuccessful, airway management based on the difficult airway algorithm was maintained.^17^ As a third method, both devices were used for intubation together with a fiberoptic bronchoscope.

For both groups, endotracheal tube was confirmed using capnography and breathing sounds. In females, the trachea was intubated with a 7.5–8.5 mm endotracheal tube, whereas for males, it was around 8.5–9.5 mm. Following the tracheal intubation, the lungs were mechanically ventilated for the whole operation period, and inhalation anesthesia was ensured with sevoflurane (1.25–1.75%) in a mixture of nitrous oxide and oxygen with a ratio 2:1. No additional medicine or treatments were applied pending the 5 minutes information collecting term after the tracheal intubation. Afterward, the administration of anesthesia was left to the anesthetist.

### Outcome measures

During each procedure, a range of data was recorded. But the most significant data collected were the rate of accomplished settlement of the endotracheal tube in the trachea as a primary outcome and the duration of the tracheal intubation process as a secondary outcome. The duration of the intubation attempt was defined as the time during which the endotracheal tube was visually confirmed to pass between the vocal cords by a qualified anesthetist. However, in cases where the endotracheal tube could not be seen passing through the vocal cords, intubation was not noted before the endotracheal tube was connected to the anesthetic circuit and the presence of end-tidal carbon dioxide was confirmed.

In addition, following data were recorded as exploratory outcome; the duration of the first unsuccessful attempt, the CL grade with the chosen device, whether the chosen device was successful, the count of initiatives, the count of successful initiatives, the time of the successful intubation process, whether alternative methods were used, the type of alternative method preferred, the need for auxiliary maneuvers or a stylet, the size of the tube, whether a difficult mask was present, any problems encountered during extubation, the pressure exerted on teeth (mild, moderate, severe), and the lowest SpO_2_ value obtained throughout all procedures. Hemodynamic changes were then evaluated. Systolic, diastolic, and mean arterial pressures, heart rate, and SpO_2_ and CO_2_ values were recorded at the time of before induction, after induction, before intubation, after the attempt with direct laryngoscope, and at the 1^st^, 3^rd^, and 5^th^ minute after the successful intubation. Data concerning the physical characteristics of patients included in this study were also recorded. This information included the following: the mandibular–mentum distance, the thyromental distance, the inter-incisor interval if jaws orifice was limited, the Mallampati score, neck movements (normal, limited, absent), the mandibular structure, tooth structure, the presence of a high larynx, a story of difficult intubation, any previous operations in the head and neck region, a history of radiotherapy, any systemic disease that could lead to difficult intubation, trauma, a history of tracheostomy, obesity, and the presence of a short neck.

### Sample size estimation

A priori sample size assessment was carried out simulating an analysis of variance (ANOVA) model in order to the duration calculations. Using two devices, an effect size of 5 seconds from our clinical knowledge, we estimated that a total of 50 patients would be needed to detect a difference between groups, with a two-tailed α of 0.05 and a (1-β) of 0.95, for a comparison of 2 independent proportions in the composite outcome measure.

### Randomisation

Patients enrolled in the study were randomly assigned in 1:1 ratio by a resident physician using an application of number and letter random generator. All data were collected by a technician who was unaware of the study groups and the type of device used in the intubation procedure.

### Statistical analysis

Data and information obtained in this present research was evaluated using Statistical Package for the Social Sciences, version 17.0. To evaluate the datum, in addition to detailed statistical techniques (mean and standard deviations), cross tabulations were used. Chi-square test and *t*-tests were applied to evaluate any differences between the groups in terms of patients’ physical characteristics (gender, age, BMI, thyromental distance, CL scores). Our primary endpoint was the success rate of endotracheal intubation in patients identified to be difficult to intubate. We used Chi-square test for the primary endpoint. Our secondary endpoint was the duration of the tracheal intubation process and we used Mann-Whitney *U* test for the analysis. The distinction in the dependent parameters of intubation attempts, intubation times, use of other tools, and overall intubation success rates for the study groups were assessed as exploratory outcomes, employing Kruskal-Wallis nonparametric one-way ANOVA and Bonferroni correction for various hypothesis testing. All detections were assessed at a 95% Confidence Interval and a *p* < 0.05 significance level.

## Results

**Figure 1:**
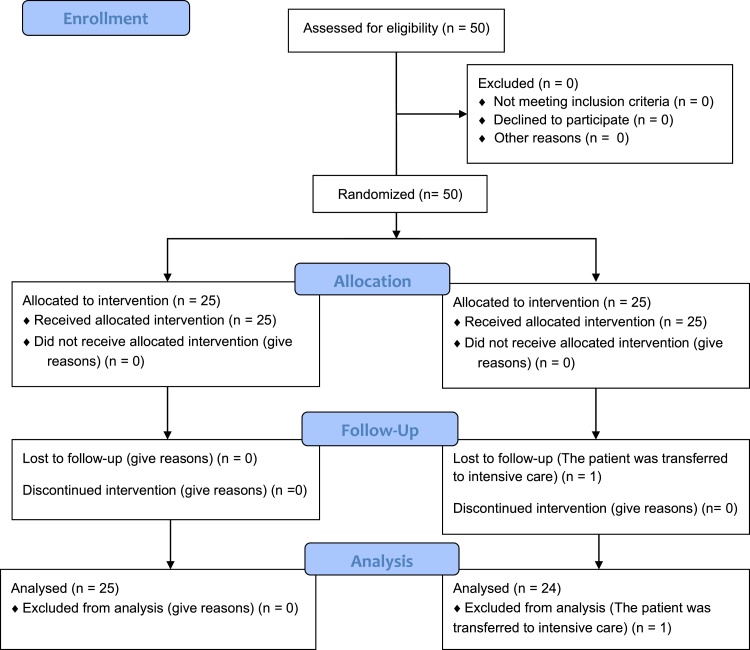
Consort flow diagram.

In total, 49 cases were included in this study: 25 cases in Group IC and 24 cases in Group VL (Fig. 1). One patient in Group VL was excluded from the study because he was transferred to the intensive care unit in the post-op period without extubation. Eligible patients were recruited to the study from January 2015 to August 2017.

No significant statistical difference was noted between the two groups in terms of age, sex, height, weight, or Body Mass Index (BMI) (*p* > 0.05) (Table 1). There were also no clinically significant group variations in terms of anesthetic management.

**Table 1:** Comparison of demographic findings according by groups.

	Group IC	Group VL	*p*
**Female**	6	6	0.935
**Male**	19	18	0.935
**Age (year)**	46.68 ± 11.327	49.45 ± 16.354	0.491
**Body height (cm)**	173.724 ± 8.857	172.25 ± 11.482	0.617
**Body weight (kg)**	83.4 ± 18.903	78.83 ± 19.977	0.415
**BMI(kg.m^2^)**	27.66 ± 5.997	26.658 ± 6.924	0.588

Data are means (SD) or numbers.

Group IC; The Intubating Catheter Group; Group VL, The Videolaryngoscope Group; BMI, Body Mass Index.

No significant statistical difference was determined among groups in terms of thyromental distance, mandibular-mentum distance, or distance between incisors (*p* > 0.05) (Table 2). Furthermore, no significant statistical difference has been identified among groups in terms of Mallampati score, the presence of small mandibula, high larynx, short and thick neck, difficult intubation history, scar tissue on the neck, or the use of auxiliary maneuvers (*p* > 0.05) (Table 2).

**Table 2:** Comparison of difficult intubation criterion by groups.

		Group IC	Group VL	*p*
		(n = 25)	(n = 24)	
**Mallampati**	1	1	0	0.458
	2	9	7	
	3	10	8	
	4	5	9	
**Thyromental distance (cm)**		7.38 ± 0.916	7.52 ± 1.433	0.683
**Interincisor distance (cm)**		4.44 ± 1.285	4.35 ± 1.440	0.827
**Mandibular corner-mentum distance (cm)**		11.6 ± 1.01	11.14 ± 1.026	0.125
**Small chin**		9 (36%)	3 (12.5%)	0.056
**Prominent upper incisor tooth**		5 (20%)	4 (16.7%)	1.000
**High larynx**		13 (52%)	10 (41.7%)	0.469
**Short-thick neck**		3 (12%)	3 (12%)	0.957
**Difficult intubation story**		2 (8%)	2 (8.3%)	0.966
**Scar tissue in neck**		2 (8)	5 (20.8)	0.382
**Extension of neck**	Adequate	22 (88%)	17 (70.8%)	
	Limited	2 (8%)	6 (25%)	
	No extension	1 (4%)	1 (4.2%)	

Data are means (SD), numbers or percentages (%).

Group IC, Intubating Catheter Group; Group VL, Videolaryngoscope Group.

The rate of successful intubation has been found to be greater in Group IC than in Group VL. However, no significant statistical difference was found (88% vs. 66%) (*p* = 0.074) (Table 3). Although the time to successful intubation was determined to be greater in Group VL than Group IC, no significant statistical difference was found (42.5 s vs. 38.6 s) (*p* = 0.593) (Table 3).

**Table 3:** Summary results for each study group (binary outcomes).

Primary outcome	Group IC	Group VL	*p*
	(n = 25)	(n = 24)	
Success rate with selected device	22/25 (88%)	16/24 (66%)	0.074
Success rate in 1^st^ attempt	16 (64%)	12 (50%)	0.322
Success rate in 2^nd^ attempt	3 (12%)	4 (16.7%)	0.953
Success rate in 3^rd^ attempt	3 (12%)	0	0.235
1^st^ trial duration (s)	51.12 ± 33.18	44.62 ± 24.89	0.528

Data are mean (SD), numbers of patients or percentages (%).

Group IC, Intubating Catheter Group; Group VL, Videolaryngoscope Group.

However, a statistical significance was observed in terms of the CL grade obtained in Group IC and in Group VL (*p* < 0.001) (Table 3). Group VL was found to have a significantly reduced CL grade. Similarly, no significant statistical difference was found among groups in terms of pressure exerted on teeth or complications. Neither there was any significant difference in terms of the number of attempts with the chosen device, the number of patients needing auxiliary maneuvers with the chosen device, the development of spasms during extubation, or the lowest SpO_2_ rates (Table 4).

**Table 4:** Reporting of summary results for each study group (continuous outcomes).

		Group IC	Group VL	*p*
		(n = 25)	(n = 24)	
**Difficult mask ventilation**		6 (24%)	8 (33%)	0.470
**Pressure applied to the tooth**	Low	2 (8%)	3 (12.5%)	0.826
	Moderate	12 (48%)	12 (50%)	
	High	11 (44%)	9 (37.5%)	
**Complication**	Yes	9	8	0.921
	No	16	16	
**Mean number of attempts with selected device for successful intubation**		1.40	1.25	0.448
**CL score with selected device**	2	1	17	0.001
	3	22	7	
	4	2	0	
**Number of patients in whom the assist maneuver was used for successful intubation**		21 (84%)	22 (91.7%)	
**Successful intubation duration with the selected device (s)**		38.68 ± 16.1	42.5 ± 27.22	0.593
**Success rate with 2^nd^ device**		2/3 (66%)	5/8 (62.5%)	
**Intubation duration with 2^nd^ device (s)**		55.5 ± 3.53	43.6 ± 16.97	
**Number of patients in whom successful intubation was achieved by the 3^rd^ method**		1 (4%)	3 (12.5%)	
**Number of patients in whom spasm developed with extubation**		1 (4%)	2 (8.3%)	0.609
**Lowest SpO_2_ value (%)**		91.4 ± 7.36	93.6 ± 3.22	0.260

Data are mean (SD), numbers or percentages (%).

Group IC, Intubating Catheter Group; Group VL, Videolaryngoscope Group; CL, Cormack-Lehane.

The success rate on the first initiative has been determined to be greater in Group IC compared to that in Group VL, but the success rate on second attempt was observed greater in Group VL than in Group IC. No significant statistical difference was found between the two groups in terms of the success rates at the first, second, and third attempts (*p* > 0.05) (Table 3). In cases wherein the initial device was replaced with the other device upon failure, five of the eight cases in Group VL were successfully intubated using Frova IC, whereas two of the three patients in Group IC were successfully intubated using the McGrath VL. The remaining four patients were intubated with both the McGrath VL and the Frova IC (Table 4).

## Discussion

In our study, the successful intubation rate using Frova IC (88%) in patients diagnosed with difficult intubation during laryngoscopy was found to be greater compared with the successful intubation rate using McGrath VL (66%).

Many studies have been performed to examine the success of ICs and VLs in difficult intubation.^10^, ^11^, ^12^, ^18^, ^19^, ^20^ Although their use is recommended in difficult airway guidelines,^21^ the number of studies examining patients diagnosed with difficult intubation in the clinic is quite limited.^12^, ^15^, ^16^ We planned our study both because we think that there is a deficiency in the literature on this subject, and because we think that a study to be conducted on patients diagnosed with difficult intubation in the clinic will be more objective than simulations performed on a manikin.

In the study by Janakiraman et al.,^22^ using a difficult intubation manikin and comparing the Frova IC with four tube changers, the success rate of the Frova IC was found to be the highest, at 78%. However, the anesthetists participating in their study had a minimum of 1 year rather than a minimum of 3 years, of anesthesia experience. In this study, performing the intubation procedures on the manikin instead on real difficult intubation cases by less experienced anesthetists explains the success rates between the two studies; this might only suggest that the success rates in our study may be more realistic.

In another study by Hodzovic et al.,^10^ which also utilized manikins, the success rate in terms of using the Frova IC was found to be 65%. Again, the manikin used in the study had a CL grade of 3, and the experience of the anesthetists was limited to 1-year. In addition, the laryngoscope blade was fixed to the manikin, and the anesthetists were let only to manipulate the tip of the IC without making any manipulations of the manikin. However, in this current research, during the replacement of the Frova IC, additional methods of adjustment, such as a change in patient position, were utilized. The higher success rates of our study may have been associated with the criteria of at least 3-years of anesthetist experience and the employment of auxiliary maneuvers.

In another study by Hodzovic et al.,^11^ which was conducted on 203 patients, the success rate of using Frova IC was found to be 96%. Success rates were 84.2%, 12%, and 15% at the first, second, and third attempts, similar to our study. However, only 32% of patients who participated in their studies were diagnosed with difficult intubation. The high success rate of the Frova IC in the study of Hodzovic et al.^11^ may be attributed to this difference.

As per our findings, the success rate of intubation obtained by McGrath VL was 66%, while in the same study by Gómez-Ríos MÁ et al.,^23^ carried out using a difficult intubation manikin, it was found to be 98%. In their study, the condition of a difficult to intubate patient was simulated by inflating the manikin’s mouth with 25 mL of air. Anesthetists involved in this study had at least 2.5 years of experience. However, they used forceps, a stylet, or the Frova IC in 9.3% of the patients. In addition, the only criterion for difficult intubation was tongue edema, while in our study, 9 out of 24 patients in Group VL fulfilled at least 2 of the difficult intubation criteria. The difference in success rates may be attributed to these factors. Similarly, in the study of Ng I et al.,^12^ the success rate with McGrath VL was 91.6%. However, in their study, the only criterion for difficult intubation was a Mallampati score of 3 or over. In the study of Wetsch WA et al.,^13^ also performed with manikins, the success of the McGrath VL was found to be 72%. Five different VLs were compared in an emergency intubation scenario in patients with a cervical spinal injury, and McGrath VL has been determined to have the lowest rate of success. Unlike our study, an attempt exceeding a duration of 300 seconds was considered unsuccessful intubation, and a stylet was used in each case.

Another parameter evaluated in this present study was the duration required to acquire successful intubation. In Group VL, the mean duration of successful intubation (excluding failed attempts) was 42.5 s, while it was 38.68 s in Group IC. This period was 16 s for Frova IC in the study by Janakiraman et al.,^22^ which used a manikin. But they had fixed the laryngoscope blade to the manikin beforehand. In the study by Gregory et al.,^20^ again using a manikin, the mean duration was 26 s. However, the duration of an attempt was limited to 60 s. The aim was to determine the effect of limited time period on successful intubation. In this present study, three patients in Group IC were intubated in over 60 s. In the study of Gómez-Ríos MÁ et al.,^23^ the mean duration of intubation was found to be 50.7 s for McGrath VL. The use of a scenario involving a tongue with edema may have caused the insertion of the laryngoscope to be delayed. The mean intubation time was found to be 37 s in the study conducted by Wallace et al.^24^ in cases that did not meet difficult intubation criteria.

In our study, significant differences were found in terms of CL grades. Lower CL grades in Group VL than those in Group IC were interpreted that VL had the effect of reducing the CL grade. This efficiency of VLs has been shown many times in various publications.^23^, ^24^ However, this advantage does not mean that the use of a VL is invariably successful in difficult to intubate patients. Although the quality of the image has increased, according to many clinicians and our own experiences, difficulties are experienced in directing the tube. For this reason, some VL firms have incorporated the stylet into the device. There was no such stylet in the McGrath VL device employed in this present study. Actually, in this present study, when there was a failure with both devices separately, the Frova IC and the McGrath VL device were used in combination; all four of the cases treated in this way were successfully intubated in a mean of 38 s. This period is shorter than the mean periods of devices separately, but a statistical comparison is not possible owing to the little instance size. Another result obtained in this present study was that the combined use of these two devices yields better results than when one is used alone. A meta-analysis has resulted in this supporting this result.^25^

In this present study, small complications such as bleeding and mucosal injury occurred during intubation, surgery, and extubation. In addition, teeth complications developed in two patients and laryngospasm developed in three patients during extubation. No significant difference was found among the two groups in terms of complication rates. In the literature, it has been demonstrated that the Frova IC poses a higher risk of tracheal or esophageal injury complications compared to other tube changers.^10^, ^11^ However, in this present study, no tracheal or esophageal injury complications occurred in association with Frova IC.

Since it is not possible to exclude the intubation skill and habit of the anesthesiologist performing the intubation procedure and it is not possible to blind the clinician, there is a possibility of bias towards the device used. In addition, the fact that CL rating is a subjective criterion is one of the limitations of the studies on this subject.

As a result of our study, the probability of successful intubation rates for Frova IC was found to be higher than McGrath VL, in line with our clinical experience. Although this difference was not statistically significant, it was a clinically significant difference. This clinical difference between the two instruments may be significant for facilities with limited financial means, given the 15-fold difference in cost in favor of Frova IC, in which case Frova IC may be preferred. In addition, practicing the use of intubation catheters, especially for clinicians with little intubation skills and experience, can be lifesaving in case of difficult intubation. However, it should not be forgotten that although both devices have advantages over each other, it should be kept in mind that the different anatomical features of the patients can change the difficult airway management.

## Funding

This work was developed at Selcuk University Faculty of Medicine, Department of Anesthesiology and Reanimation, Konya, Turkey. This paper was not presented at any congress or conference. But this study was presented at Selcuk University Faculty of Medicine, Department of Anesthesiology and Reanimation as a thesis on December 2017 by Dr. Aysun Ozdemirkan.

## Conflicts of interest

The authors declare no conflicts of interest.

## Funding

This clinical trial was funded by Selcuk University Scientific Research Office under number “15102023”.
